# The gut-prostate axis in benign prostatic diseases: Mechanistic pathways and therapeutic implications

**DOI:** 10.1016/j.isci.2026.117322

**Published:** 2026-08-28

**Authors:** Qizhen Tang, Kenan Wang, Weiwei Fan, Quanxin Su

**Affiliations:** 1 Department of Urology, The First Affiliated Hospital of Dalian Medical University, Dalian, Liaoning, China

**Keywords:** gut microbiota, benign prostatic hyperplasia, chronic prostatitis, gut-prostate axis, interventions

## Abstract

Benign prostatic hyperplasia (BPH) and chronic prostatitis/chronic pelvic pain syndrome (CP/CPPS) are common benign prostatic diseases in middle-aged and older men. The prevalence of BPH increases with age, affecting approximately 45% of men older than 45 years and nearly 80% of those older than 70 years. CP/CPPS predominantly affects men aged 30–50 years, with a global prevalence of approximately 8%. Conventional pathogenic models emphasize androgen metabolism, chronic inflammation, aging, and genetic susceptibility; however, these factors do not fully explain the marked clinical heterogeneity of these disorders. Recent evidence suggests that the gut microbiota may influence prostatic inflammation and hyperplasia through microbial metabolites, immune regulation, and neuroendocrine pathways, giving rise to the concept of a gut-prostate axis. Animal experiments and clinical association studies have reported significant differences in gut microbial composition between patients with BPH or CP/CPPS and healthy controls, and some studies have suggested that microbiota-directed interventions, such as fecal microbiota transplantation, may have potential for alleviating pelvic pain. This narrative review selectively synthesizes evidence from published systematic reviews and original studies to discuss the relationship between the gut microbiota and BPH and CP/CPPS, evaluate the proposed mechanisms and current controversies, and outline future research directions and translational prospects.

## Graphical abstract

**Figure:**
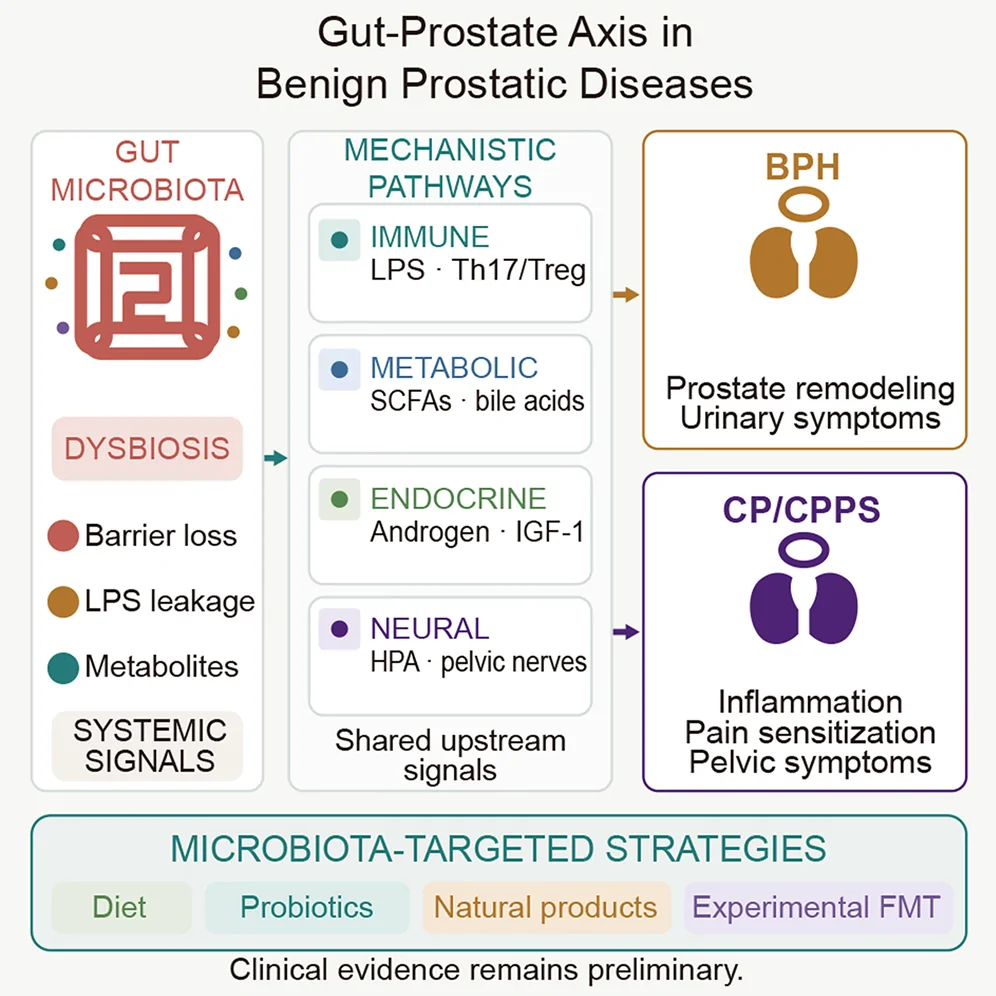

## Teaser

Health sciences

## Introduction

Benign prostatic diseases are among the most common chronic conditions encountered in male urology and include benign prostatic hyperplasia (BPH) and chronic prostatitis/chronic pelvic pain syndrome (CP/CPPS). BPH is an important cause of lower urinary tract symptoms (LUTS) in older men, and its prevalence increases progressively after the age of 40 years.^1^ In 2019, approximately 79 million men aged 60 years or older were estimated to have BPH, making it a major public health issue that impairs urinary function and quality of life in aging men.^2^ CP/CPPS is characterized primarily by chronic pelvic, perineal, or prostatic pain, often accompanied by variable urinary symptoms and sexual dysfunction. It is the most common and most difficult-to-treat form of chronic prostatitis, and no universally effective treatment strategy has been established.^3^

Current research on the conventional pathogenesis of BPH and CP/CPPS has focused mainly on stromal-epithelial interactions, chronic inflammation, oxidative stress, genetic susceptibility, and neuroendocrine abnormalities.^4^^,^^5^ However, these mechanisms do not fully account for clinical features such as symptom heterogeneity, recurrent disease, and poor responses to therapy in some patients. In CP/CPPS in particular, pain, urinary symptoms, and psychological disturbances interact in a complex manner, and symptoms may arise from the combined effects of immune, neural, endocrine, and psychological factors.^6^ Expanding the focus from local prostatic lesions to systemic metabolic-immune-neural networks may therefore provide a new explanatory framework for benign prostatic diseases.

The gut microbiota is a complex microbial ecosystem inhabiting the gastrointestinal tract. Its composition is shaped by diet, age, medication exposure, immune status, and the metabolic environment, and it remains in dynamic equilibrium with the host.^7^ As an important metabolic and immune organ, the gut microbiota participates in the development and progression of multiple chronic diseases by producing short-chain fatty acids (SCFAs), regulating bile acid and tryptophan metabolism, maintaining the intestinal mucosal barrier, shaping immune homeostasis, and modulating neuroendocrine networks.^8^^,^^9^^,^^10^ With the development of concepts such as the gut-brain axis, gut-bladder axis, and gut-urogenital axis, the link between the gut microbiota and prostatic diseases has attracted increasing attention. A study by Meng et al. provides a comprehensive overview of the gut-prostate axis in chronic prostatitis, BPH, and prostate cancer.^11^ Existing studies have shown decreased gut microbial diversity in patients with CP/CPPS and associations between the Firmicutes/Bacteroidetes ratio, specific enterotypes, selected bacterial genera, prostate enlargement, and disease risk in patients with BPH.^12^ Nevertheless, this field remains at an early stage. Key pathogenic and protective taxa, core metabolites, causal relationships, and translatable therapeutic targets remain unclear. This review therefore summarizes the evidence linking the gut microbiota to benign prostatic diseases, examines potential mechanisms, and discusses clinical translational value, with the aim of informing mechanistic interpretation, risk stratification, and microbiota-based intervention strategies for BPH and CP/CPPS.

## Associations between the gut microbiota and benign prostatic diseases

### BPH

#### Microbial diversity changes in patients with BPH

Findings regarding gut microbial diversity in patients with BPH are not entirely consistent. Table S1 summarizes 12 original clinical studies of BPH/LUTS and CP/CPPS, including the study population, sample size, analytical method, alpha- and beta-diversity findings, and the principal differential taxa, metabolites, or functional features. Tsai et al. compared fecal and urinary microbiota among patients with BPH, patients with prostate cancer, and controls, and found that between-group differences were less apparent in fecal microbiota than in urinary microbiota.^13^ Thus, not all patients with BPH necessarily exhibit marked global fecal dysbiosis, and gut microbial signals may be influenced by sample source, disease phenotype, and population background. In contrast, Ratajczak-Zacharko et al. conducted enterotype analysis in older men and found differences in beta diversity and enterotype distribution between patients with BPH and healthy controls; enterotypes characterized by *Blautia*, *Bacteroides*, and *Streptococcus* were associated with BPH.^14^ These findings suggest that BPH-related microbial alterations may not be reflected simply by a unidirectional increase or decrease in alpha diversity, but rather by rearrangement of community structure and ecological types. Using prostate volume as the entry point, Takezawa et al. found that men with prostate enlargement had an increased Firmicutes/*Bacteroidetes* ratio, accompanied by increased Firmicutes and Actinobacteria and decreased *Bacteroidetes*.^15^ Although the Firmicutes/*Bacteroidetes* ratio is a relatively crude ecological indicator, it has been linked to energy metabolism, obesity, and low-grade inflammation. Overall, current evidence supports the presence of structural shifts in the gut microbiota of patients with BPH rather than a simple decline in microbial diversity. In general, studies of the gut microbiota in BPH indicate unstable changes in overall diversity but more prominent differences in beta diversity and specific microbial taxa.

#### Associations between the gut microbiota and clinical indicators of BPH

Compared with overall diversity, associations between the gut microbiota and clinical indicators of BPH may offer greater explanatory value. In men with LUTS, Holland et al. found that multiple fecal operational taxonomic units were associated with different components of the International Prostate Symptom Score (IPSS), with Lachnospiraceae, *Blautia*, and related taxa associated with lower symptom bother.^16^ These findings suggest that the gut microbiota may not only be associated with disease status but may also contribute to symptom severity and heterogeneity. Studies of microbial metabolites further extend this concept. Ratajczak et al. reported altered fecal profiles of SCFAs and branched-chain fatty acids (BCFAs) in men with BPH, including increased isobutyric and isovaleric acids, whereas isohexanoic acid was higher in healthy controls.^17^ Their subsequent work linked SCFA/BCFA profiles to metabolic syndrome, lipid parameters, and prostatic tissue expression of IL-6 and IL-18.^18^ These findings suggest that the relationship between BPH and the gut microbiota may operate primarily through a microbiota-metabolism-metabolic syndrome-prostatic inflammation pathway rather than through compositional differences alone. In recent years, associations between specific taxa and clinical indicators have also gained attention. Wu et al. found reduced *Akkermansia* abundance in patients with BPH, which was associated with IPSS, body mass index, glucose levels, and lipid parameters.^19^ Lin et al., integrating Mendelian randomization, clinical retrospective analysis, and fecal 16S sequencing, reported increased Escherichia-Shigella in patients with BPH, an association with severe LUTS, and potential protective signals for *Lactobacillus* and related taxa.^20^ These findings further link BPH-associated microbial changes to the intestinal barrier, metabolic syndrome, and symptom severity. In patients with BPH stratified by prostate volume, Liu et al. found no significant differences in overall alpha or beta diversity, but differential taxa and metabolites were involved in unsaturated fatty acid, amino acid, and steroid hormone biosynthesis pathways and were associated with prostate volume, prostate-specific antigen (PSA), and related indicators.^21^ Surber et al. also found that, among patients with BPH/LUTS undergoing transurethral surgery, certain microbial features were associated with prostate volume and postvoid residual urine, including decreased *Methanobrevibacter smithii* in the large-prostate group and decreased *Collinsella* together with increased Gastranaerophilales in patients with high postvoid residual urine.^22^

### CP/CPPS

Studies of the gut microbiota in patients with CP/CPPS are fewer than those in BPH, but available clinical evidence suggests possible dysbiotic features. Shoskes et al. compared fecal microbiota from 25 patients with CP/CPPS and 25 healthy controls and found significantly reduced alpha diversity in the CP/CPPS group. UniFrac principal coordinate analysis showed clear separation between groups, and decreased Prevotella and related taxa were observed, indicating altered gut microbial diversity and composition in CP/CPPS.^23^ Wang et al. enrolled 41 patients with CP/CPPS and 43 healthy controls and analyzed the gut microbiota using fecal 16S rRNA sequencing. They identified significant compositional differences between patients and controls and developed a gut microbiota-based disease prediction model.^24^ Their results showed reduced abundance of *Blautia*, *Faecalibacterium*, *Bifidobacterium*, *Prevotella 9*, *Alistipes*, *Dialister*, and related taxa in CP/CPPS, whereas *Bacteroides*, *Romboutsia*, *Escherichia-Shigella*, *Ruminococcus*, *Enterobacter*, *Pseudomonas*, *Clostridium sensu stricto 1*, *Lachnoclostridium*, and related taxa were increased.

Animal studies provide complementary evidence for gut dysbiosis in CP/CPPS. In a rat model of chronic prostatitis-like inflammation, Konkol et al. observed reduced *Lactobacillus*, Lachnospiraceae, and *Bacteroides uniformis*, along with increased *Odoribacter*, Clostridiaceae, and Rikenellaceae.^25^ Du et al. found alterations in gut microbial structure and SCFA levels in a model of experimental autoimmune prostatitis (EAP).^26^ Together with clinical observations, these animal studies support a consistent association between CP/CPPS and gut microbial dysbiosis.

Compared with cross-sectional microbiome comparisons, Mendelian randomization studies provide stronger human genetic evidence regarding the direction of association between selected gut microbial taxa and prostatic diseases. Previous MR studies have reported genetically predicted associations between specific taxa and BPH. More comprehensively, Liu et al. evaluated 196 gut microbial taxa in relation to prostate cancer, prostatitis, and BPH using two-sample MR, reverse MR, linkage disequilibrium score regression, colocalization analysis, and two-step mediation analysis. Across the three disease groups, they identified 31 taxa with putative causal associations, 53 candidate mediators involving sex hormones, circulating metabolites, and proteins, and 34 candidate protein targets, including FGFR1 and XPNPEP1. Ruminococcaceae UCG009 showed a protective genetic association with prostatitis, whereas Clostridiales was associated with an increased risk of BPH. These findings strengthen the genetic support for a gut–prostate relationship but should be interpreted cautiously. The genome-wide association study (GWAS)-defined prostatitis phenotype may not correspond specifically to National Institutes of Health (NIH) category III CP/CPPS.^27^

## Potential mechanistic pathways linking the gut microbiota to the prostate

Although BPH and CP/CPPS are discussed within a common gut-prostate framework, they are pathophysiologically distinct conditions. In this section, the gut-prostate axis refers only to potentially shared gut-derived upstream influences, including microbial metabolites, systemic inflammation, metabolic alterations, and neuroendocrine signaling. It does not imply that BPH and CP/CPPS share identical downstream mechanisms. Accordingly, evidence is interpreted separately for each condition: BPH-related evidence is considered mainly in relation to androgen-sensitive growth, metabolic inflammation, and tissue remodeling, whereas CP/CPPS-related evidence is considered mainly in relation to immune dysregulation, pain sensitization, and pelvic floor dysfunction.

### The intestinal barrier-pattern recognition receptor axis: An upstream trigger of chronic prostatic inflammation

**Figure 1:**
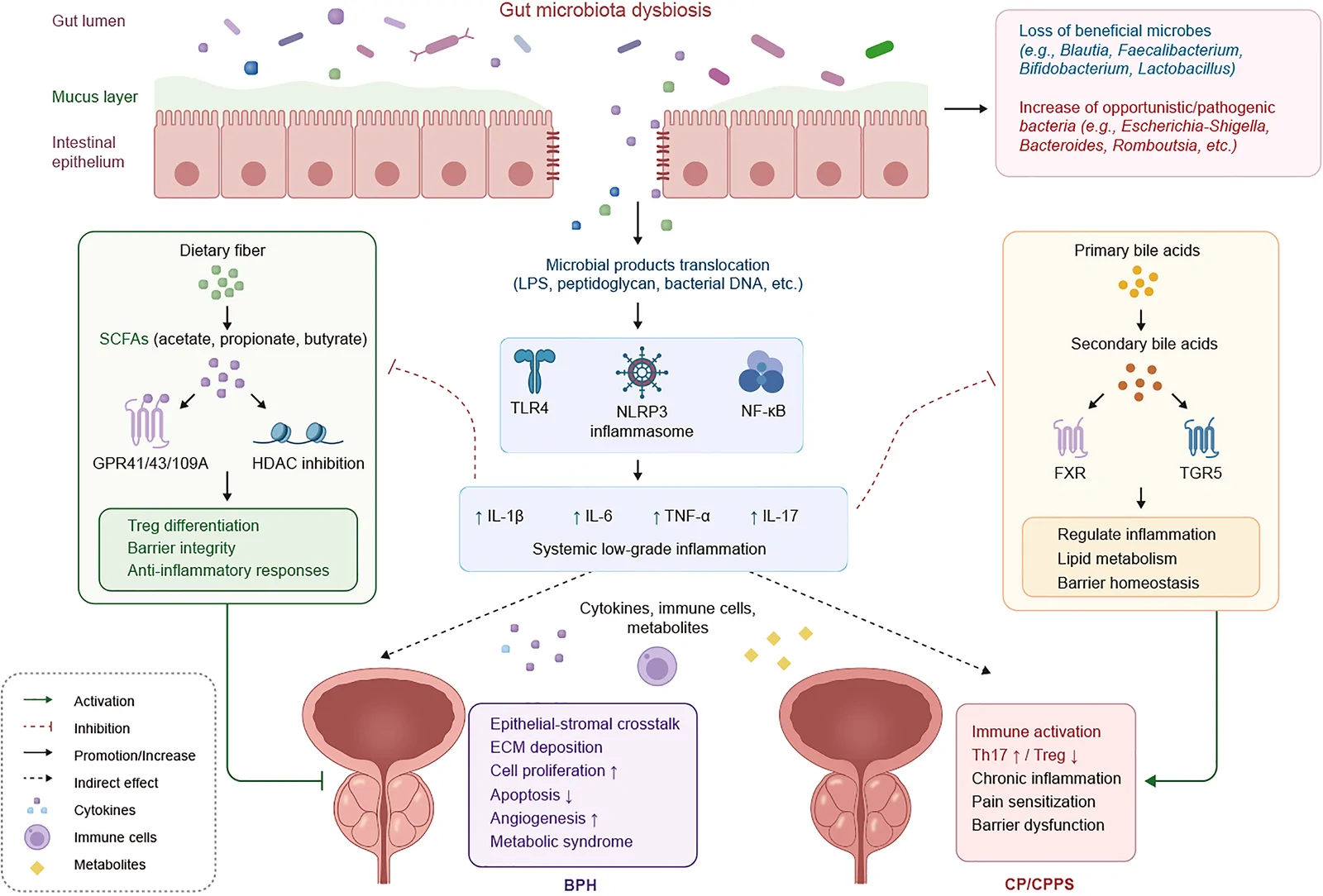
Proposed pathways linking the gut microbiota to BPH and CP/CPPS Gut dysbiosis may alter intestinal barrier integrity, microbial product translocation, immune signaling, microbial metabolites, metabolic and endocrine homeostasis, and neural-humoral communication. These upstream signals may be associated with distinct downstream responses: metabolic inflammation, androgen-sensitive growth, and epithelial-stromal remodeling in BPH, and immune dysregulation, Th17/Treg imbalance, pain sensitization, and symptom persistence in CP/CPPS.

Disruption of the intestinal barrier and subsequent activation of pattern recognition receptors may represent a primary pathway through which the gut microbiota affects prostatic disease (Figure 1). Under physiological conditions, the intestinal epithelium, mucus layer, tight junction proteins, and local immune cells collectively restrict bacterial components from entering the circulation. During dysbiosis, reductions in mucus-producing and barrier-supporting taxa, together with relative expansion of opportunistic pathogens and Gram-negative bacterial components, can increase intestinal permeability. This allows microbial-associated molecular patterns such as lipopolysaccharide (LPS), peptidoglycan, and bacterial DNA to enter the bloodstream. These signals can activate pathways such as TLR4, NF-κB, and the NLRP3 inflammasome, inducing the release of inflammatory mediators including IL-1β, IL-6, TNF-alpha, and IL-17 and thereby establishing a persistent low-grade inflammatory state.

This mechanism is particularly relevant to CP/CPPS. Patients with CP/CPPS often lack evidence of a clear pathogen-driven infection yet may exhibit chronic pain and inflammatory responses. Thus, the source of inflammation may not be confined to local prostatic infection but may also involve continuous input from gut-derived immune stimulation. In an EAP model, Du et al. found that gut dysbiosis shifted the Th17/Treg balance toward a proinflammatory state and aggravated prostatic inflammation, indicating that the gut microbiota can reshape the prostatic immune microenvironment.^26^ Ning et al. further showed that total tanshinones extract alleviated CP/CPPS-like pathological changes by modulating the gut microbiota and suppressing the LPS-TLR4 axis, providing additional support for a role of gut-derived endotoxin signaling in maintaining prostatic inflammation.^28^

In BPH, a direct intestinal barrier-inflammation pathway has not yet been established. Current BPH-specific evidence mainly supports associations among gut microbial alterations, metabolic abnormalities, systemic inflammatory status, and prostate enlargement, suggesting that gut-derived inflammatory signals may modify, rather than directly initiate, the prostatic growth environment. Persistent low-grade inflammation in the prostate can promote stromal cell activation, enhanced epithelial-stromal interactions, extracellular matrix deposition, and dysregulated proliferation and apoptosis, thereby creating a microenvironment conducive to prostatic hyperplasia. In testosterone-induced BPH rats, Li et al. found that BPH development was accompanied by changes in gut microbial composition and metabolic profiles, suggesting that the gut microbial ecology may participate in systemic inflammation and metabolic remodeling during prostate enlargement.^29^ An et al. further reported that both BPH induction and finasteride treatment altered gut microbial structure and were associated with changes in prostatic morphology, hormone-related parameters, and apoptosis markers.^30^

Taken together, the intestinal barrier-pattern recognition receptor axis is mechanistically supported mainly by studies of CP/CPPS-related inflammation and experimental prostatitis models. In BPH, gut-derived inflammatory signals may plausibly modify the epithelial, stromal, and vascular microenvironment; however, direct evidence demonstrating intestinal barrier disruption, microbial translocation, and pattern recognition receptor activation in human BPH remains limited.

### The microbial metabolite-immunometabolic axis: From SCFAs to bile acids and lipid signals

Unlike the inflammatory axis, the metabolite axis emphasizes how the gut microbiota alters host immunity, metabolism, and tissue homeostasis through absorbable small-molecule signals. SCFAs, including acetate, propionate, and butyrate, are the most extensively studied microbial metabolites. They are not only products of dietary fiber fermentation but also important immunometabolic signals that regulate T cell differentiation, macrophage polarization, barrier repair, and inflammatory cytokine expression through SCFA-sensing G protein-coupled receptors, including GPR41, GPR43, and GPR109A, as well as histone deacetylase-dependent pathways.^26^^,^^31^^,^^32^

In CP/CPPS, preclinical studies have provided relatively detailed evidence linking SCFA alterations to immune dysregulation. In an EAP model, Du et al. found that gut dysbiosis reduced propionate levels, which in turn impaired Treg differentiation and enhanced Th17 responses through a GPR43-HDAC6-related mechanism, ultimately sustaining prostatic inflammation. Propionate supplementation partially corrected Th17/Treg imbalance and reduced disease susceptibility.^26^ The significance of this finding lies in its conversion of microbial compositional change into a mechanistic explanation in which deficiency of a specific metabolite impairs immune tolerance. In addition, Guan et al. reported that activation of the IGF1/IGF1R-PKC-β pathway in the EAP mouse model aggravated prostatic inflammation and pain, promoted Th17 differentiation, and reduced the Treg proportion, whereas Igf1r knockdown alleviated the inflammatory phenotype.^33^ In their study of alcohol intake and CP/CPPS, Liu et al. also found that alcohol-associated symptom aggravation was linked to reductions in microbiota-derived propionate and butyrate, suggesting that decreased SCFAs may simultaneously impair the intestinal barrier and immune tolerance, thereby amplifying prostatic inflammation.^34^

In BPH, SCFA-related mechanisms appear to be expressed mainly through metabolic homeostasis. BPH is closely associated with obesity, insulin resistance, and metabolic syndrome, whereas SCFAs can influence energy harvest, intestinal barrier function, lipid metabolism, insulin sensitivity, and systemic inflammation. Liu et al. found that the gut microbiota and its metabolites were associated with prostate volume and symptoms in patients with BPH, involving pathways related to unsaturated fatty acids, amino acids, and steroid hormone biosynthesis.^21^ These findings indicate that BPH-related microbial alterations should not be interpreted solely as taxonomic differences; instead, their metabolic functions and potential contribution to a metabolically inflamed prostatic microenvironment should be considered.

Beyond SCFAs, bile acids and lipid metabolism may also serve as important links between the gut microbiota and the prostate. Gut microbes convert primary bile acids into secondary bile acids and regulate inflammation, lipid metabolism, and barrier homeostasis through receptors such as farnesoid X receptor (FXR) and TGR5. In EAP, antibiotic cocktail-induced microbial remodeling altered the bile acid profile and restrained Th17 differentiation through the FXR-NLRP3 axis, thereby alleviating prostatic inflammation.^35^ Conversely, alcohol-related EAP studies suggest that microbiota-driven enhancement of cholesterol biosynthesis promotes Th17 differentiation and worsens prostatic inflammation.^36^ These findings collectively indicate that microbial metabolites are not intrinsically anti-inflammatory or proinflammatory; rather, their effects depend on the specific metabolite, receptor pathway, and host immune context.

In summary, the core of the metabolite axis is not simply the increase or decrease of a particular bacterial taxon, but the alteration of functional modules within the gut microbiota. In CP/CPPS, available evidence links changes in SCFAs, bile acid signaling, and lipid metabolism to Th17/Treg imbalance and inflammatory responses. In BPH, current evidence is primarily associative and links microbial metabolites to metabolic syndrome, steroid-related metabolic pathways, prostate volume, and LUTS. A causal metabolite-driven mechanism for prostatic hyperplasia has not yet been established.

### The endocrine-growth factor axis: Microbial remodeling of the prostatic proliferative environment

Endocrine regulation is among the most specific mechanisms in BPH and represents an important route through which the gut microbiota may influence prostatic hyperplasia. The development and progression of BPH depend on androgen signaling, particularly the conversion of testosterone to dihydrotestosterone (DHT) and activation of the androgen receptor. At the same time, insulin, insulin-like growth factor 1 (IGF-1), inflammatory mediators, and lipid metabolic abnormalities may jointly promote proliferation of prostatic epithelial and stromal cells. The gut microbiota may indirectly reshape the endocrine and growth-factor milieu of the prostate by regulating bile acid metabolism, enterohepatic circulation of steroid hormones, insulin sensitivity, and systemic inflammation.

In testosterone-induced BPH rats, Li et al. found that prostatic hyperplasia was accompanied by changes in gut microbial diversity, composition, and metabolites, suggesting that exogenous androgen-driven prostate enlargement is not an isolated local event but may occur in parallel with systemic microbial ecological and metabolic changes.^29^ An et al. showed that finasteride, while improving BPH-related prostatic morphology and hormone-related parameters, also reshaped the gut microbiota and was associated with apoptosis-related indicators in prostatic cells. These findings suggest that the effects of 5α-reductase inhibitors may extend beyond inhibition of DHT production and may involve changes in host-microbiota metabolic networks.^30^ Mechanistically, the microbiota may not directly determine intraprostatic DHT levels; instead, it may enhance prostatic sensitivity to androgens and growth factors through metabolic syndrome and low-grade inflammation. The concept of metabolic endotoxemia proposed by Cani et al. indicates that gut-derived LPS can drive low-grade inflammation, weight gain, and insulin resistance.^37^ Insulin resistance and enhanced insulin growth factor-1 (IGF-1) signaling can in turn provide a growth-promoting environment for prostatic cells. In a population-based multi-omics study, Liu et al. found that microbial and metabolic changes in patients with BPH involved steroid hormone biosynthesis and prostate volume-related indicators, providing indirect support for the microbiota-metabolic endocrine-prostatic proliferation hypothesis.^21^

For CP/CPPS, endocrine mechanisms are not considered the dominant cause but may contribute to disease maintenance. Chronic pain, anxiety, inflammation, and sleep disturbance can affect the hypothalamic-pituitary-adrenal (HPA) axis and sex hormone status, whereas the gut microbiota may regulate stress responses through inflammatory mediators, SCFAs, and bile acid metabolism. Liu et al. reported that the beneficial effects of *Poria cocos* (*P. cocos*) polysaccharides in chronic nonbacterial prostatitis may involve oxidative stress, hormone production, and gut microbial alterations, suggesting that, in CP/CPPS, endocrine regulation is more likely to act as a downstream component or amplifier of the inflammation-stress network.^38^

Thus, the endocrine-growth factor axis is most directly relevant to BPH, in which androgen signaling, insulin resistance, IGF-1 activity, and metabolic inflammation may influence epithelial and stromal proliferation. In CP/CPPS, endocrine and stress-related alterations may modify symptom persistence or inflammatory responses, but they are not established as primary disease-driving mechanisms. The key issue is not whether a single genus directly increases or decreases androgen levels, but whether the gut microbiota, through metabolic inflammation and the hormonal metabolic background, alters the responsiveness of prostatic tissue to androgens, growth factors, and inflammatory stimuli.

### The neural-humoral axis: Gut-brain axis-mediated pelvic nerve sensitization, pelvic floor hypertonicity, and urinary dysfunction

In addition to inflammatory and metabolic mechanisms, the gut microbiota may amplify prostatic disease-related symptoms through a gut-brain-prostate axis, particularly chronic pelvic pain and pelvic floor hypertonicity in CP/CPPS and urinary dysfunction in BPH/LUTS. The gut microbiota can affect central pain processing and autonomic activity through the enteric nervous system, vagus nerve, sympathetic and parasympathetic pathways, the HPA axis, and microbial metabolites. Conversely, chronic pain, anxiety, and stress can alter intestinal motility, barrier function, and microbial composition, forming a positive feedback loop of dysbiosis, stress response, neural sensitization, and symptom persistence.^39^^,^^40^^,^^41^ In an EAP mouse model, abnormal gut microbial composition was associated with prostatitis-related depressive-like behaviors.^42^ In patients with CP/CPPS, Kong et al. found that symptom improvement following low-intensity extracorporeal shock-wave therapy was accompanied by changes in gut microbial diversity and predicted functional pathways.^43^ This mechanism helps explain the frequent mismatch between the degree of local inflammation and pain intensity in patients with CP/CPPS.

In CP/CPPS, the possible role of the gut-brain axis is not merely to exacerbate inflammation, but to lower the excitability threshold of the pelvic nervous system. Dysbiosis may reduce SCFAs, alter tryptophan/serotonin metabolism, affect neuroactive substances such as gamma-aminobutyric acid, and activate the HPA axis, thereby facilitating sensitization of the spinal dorsal horn and pelvic afferent nerves. Under these conditions, mild stimuli originating from the prostate, bladder neck, urethra, or pelvic floor muscles may be amplified into persistent perineal pain, urethral discomfort, ejaculatory pain, or abnormal urinary urgency. He et al. reported that CP/CPPS involves abnormalities of sensory, autonomic, and central nervous systems, and Yilmaz et al. also identified autonomic nervous system changes in men with CP/CPPS, suggesting that dysregulated neural control may be an important mechanism underlying chronic pelvic pain persistence.^44^^,^^45^

The gut-brain-prostate axis may also influence pelvic floor status through autonomic regulation. Chronic stress and sympathetic activation can place the pelvic floor muscles in a state of protective contraction, leading to pelvic floor hypertonicity, impaired relaxation, and the development of myofascial trigger points. Persistent pelvic floor tension can then increase local mechanical stimulation and nociceptive input, further exacerbating pelvic pain. Westesson et al. noted that some patients with CP/CPPS exhibit marked pelvic floor spasm. Shoskes et al. found greater abdominal and pelvic tenderness in patients with CP/CPPS than in controls. Yani et al. further reported impaired pelvic floor muscle relaxation in patients with CP/CPPS and ejaculatory pain, suggesting that pelvic floor hypertonicity may not simply be a secondary symptom but may represent an important amplifier within the neural sensitization network.^46^^,^^47^^,^^48^

This neural regulatory mechanism is supported mainly by studies of CP/CPPS and chronic prostatitis models, in which pelvic floor dysfunction and cross-organ sensitization may contribute to urinary symptoms. Although neural and bladder-related factors may also modify LUTS in patients with BPH, direct evidence linking the gut microbiota to these processes in BPH remains limited. When the pelvic floor muscles cannot relax adequately, coordination between the external urethral sphincter and pelvic floor muscles during voiding may be impaired, causing weak urinary stream, hesitancy, incomplete emptying, frequency, and urgency. He et al. found that pelvic floor biofeedback improved symptoms in patients with chronic prostatitis and dysfunctional voiding, suggesting that some urinary abnormalities are not caused solely by prostatic inflammation or mechanical obstruction but are closely related to pelvic floor dysfunction and abnormal neural regulation.^49^ In patients with BPH, prostate volume does not always parallel LUTS severity, suggesting that, in addition to gland enlargement, autonomic excitation, bladder sensory sensitization, pelvic floor discoordination, and microbiota-related metabolic or humoral signals may jointly contribute to symptom generation.^50^

Furthermore, the prostate, bladder, urethra, and pelvic floor muscles share some afferent pathways at the spinal level. Therefore, gut-brain axis-induced neural sensitization may extend to the entire prostate-bladder-pelvic floor functional unit. Schwartz et al. showed that chronic prostatitis induced bladder hypersensitivity and sensitized bladder afferents, and Aydogdu et al. proposed that prostate-to-bladder cross-organ sensitization can promote urinary frequency, urgency, and bladder discomfort.^51^^,^^52^ Additional biological plausibility is provided by studies in other urological pelvic-pain models. In AOAH-deficient mice, gut dysbiosis and altered microbial metabolites contributed to pelvic allodynia, and normalization of the microbiota alleviated the pain phenotype. Subsequent work in the same model implicated central microglial activation in pelvic pain.^53^^,^^54^ Thus, the gut microbiota does not directly cause pelvic floor spasm or voiding dysfunction, but it may amplify prostatic pain, pelvic floor hypertonicity, and urinary abnormalities by modulating central pain processing, autonomic tone, and cross-organ sensitization within the pelvis through the gut-brain-prostate axis. In contrast to the inflammatory-immune axis, which mainly explains local pathological changes, the neural-humoral axis is better suited to explaining the persistent, fluctuating symptoms of CP/CPPS that do not fully correspond to the extent of local pathology. However, its specific role in BPH requires further investigation.

## Common risk factors affecting the gut microbiota and benign prostatic diseases

### Dietary factors

Diet is an important exogenous factor that influences both gut microbial homeostasis and the progression of benign prostatic diseases. High-fat diets have relatively direct evidence in BPH-related research. Gu et al. found that a high-fat diet induced gut microbial alterations in rats and was associated with BPH progression, suggesting that excessive fat intake may contribute to the prostatic hyperplasia phenotype by altering the gut microbial ecology.^55^ In population-based studies, a higher dietary inflammatory index has been associated with increased risk or symptom burden of LUTS/BPH in men, whereas greater adherence to the Mediterranean diet has been associated with milder urinary symptoms, indicating that dietary inflammatory potential and overall diet quality may influence the BPH/LUTS phenotype.^56^^,^^57^^,^^58^

Direct evidence linking high-sugar diets to benign prostatic diseases remains limited, but their influence on the gut microbiota is supported by substantial evidence. High sugar intake can shift the gut microbiota toward a more proinflammatory state and impair the function of some beneficial taxa. Westernized diets, which are typically high in fat and sugar and low in fiber, can disrupt gut microbial structure and function.^59^^,^^60^ Direct nutrient-intake evidence has also been reported in relation to prostatitis-like symptoms. In a Chinese adult cohort of 1,284 participants, Zhang et al. found that higher intakes of fat, vitamin E-(β+γ), magnesium, sodium, and copper were associated with greater susceptibility to prostatitis-like symptoms after adjustment for potential confounders.^61^ With respect to spicy or irritant foods, Herati et al. reported that spicy foods, coffee, chili, alcohol, and tea aggravated symptoms in some patients with CP/CPPS. A case-control study from China also suggested associations between dietary and lifestyle factors and CP/CPPS as well as pain severity.^62^^,^^63^ Therefore, spicy and irritant foods are more appropriately described as symptom triggers or aggravating factors, and whether this effect is mediated by the gut microbiota requires further study.

Insufficient intake of dietary fiber and probiotics may weaken gut microbial diversity and stability. Low-fiber diets are thought to deplete microbial diversity, whereas population studies show that higher dietary fiber intake is associated with changes in gut microbial composition and increased abundance of potentially beneficial taxa such as *Faecalibacterium*.^64^^,^^65^ Wastyk et al. compared high-fiber and fermented-food dietary interventions and found that fermented foods increased gut microbial diversity and improved immune-related indicators. These findings suggest that inadequate dietary fiber, fermented foods, and probiotic sources in daily diets may be unfavorable for maintaining gut microbial homeostasis.^66^

### Lifestyle factors

Sedentary behavior and insufficient physical activity may affect benign prostatic diseases through both pelvic local conditions and intestinal function. In middle-aged Korean men, Park et al. found that longer sitting time and lower physical activity were associated with increased risk of LUTS.^67^ A prospective study by Zhang et al. showed that higher leisure-time physical activity was associated with a lower risk of CP/CPPS in middle-aged and older men.^68^ In parallel, exercise interventions and physical activity have been associated with improvements in gut microbial composition and alpha diversity. A meta-analysis of exercise interventions in adults showed an increase in the Shannon index after exercise, suggesting that physical inactivity may be unfavorable for maintaining gut microbial diversity.^69^^,^^70^

Alcohol consumption is a representative shared risk factor linking CP/CPPS and gut dysbiosis. Liu et al. found that patients with CP/CPPS who consumed alcohol had higher NIH-Chronic Prostatitis Symptom Index (NIH-CPSI) scores and abnormalities in gut microbiota and SCFAs; several indicators showed partial recovery in individuals who discontinued alcohol use, suggesting that alcohol-associated dysbiosis may be related to symptom severity.^34^ Animal studies also show that alcohol intake can aggravate EAP and is accompanied by gut microbial abnormalities.^36^ Regarding sleep loss and circadian disruption, current evidence derives mainly from gut microbiota research. Sleep deprivation and circadian disruption can induce gut dysbiosis, metabolic abnormalities, and intestinal barrier dysfunction; however, direct evidence linking these factors to BPH or CP/CPPS remains limited, and they should therefore be described cautiously as potential shared risk factors.^71^^,^^72^^,^^73^

### Age and underlying diseases

Age is one of the clearest nonmodifiable risk factors for BPH. BPH is a common disease in older men, and both histological and clinical prevalence increase with age. Classic studies also identify aging and androgens as important foundations for the development of BPH/benign prostatic enlargement (BPE).^74^^,^^75^ At the same time, aging itself is associated with changes in gut microbial structure, function, and stability. However, gut microbiota in older adults does not necessarily show a simple decline in diversity; healthy aging, diet, medication use, and lifestyle can substantially influence microbial trajectories.^76^^,^^77^ Middle-aged and older men may therefore occupy an intersecting state in which BPH risk is elevated and intestinal microecological vulnerability is increased.

Obesity, diabetes, and metabolic syndrome are important underlying conditions linking gut dysbiosis with BPH/LUTS. Metabolic syndrome is associated with increased prostate volume, PSA levels, and annual prostate growth rate in patients with BPH, and patients with diabetes have a greater BPH/LUTS symptom burden.^78^^,^^79^ A positive association between obesity and BPH/LUTS is supported by considerable epidemiological evidence, and animal studies of obesity-associated BPH show concomitant gut dysbiosis, suggesting that metabolic abnormalities may aggravate prostatic disease phenotypes through alterations in the gut microbial ecology.^80^^,^^81^ It should be noted, however, that the association between metabolic abnormalities and CP/CPPS is less consistent than that for BPH. Some cohort studies have not found significant associations between obesity, smoking, hypertension, and the risk of CP/CPPS.^82^

## Gut microbiota-based interventions for benign prostatic diseases

Overall, gut microbiota-targeted therapy for benign prostatic diseases remains exploratory. Existing strategies include probiotics and prebiotics, dietary and lifestyle regulation, fecal microbiota transplantation (FMT), microbiota modulation by traditional Chinese medicine and natural products, and clinical combination therapies.

### Probiotic and prebiotic interventions

Probiotic intervention is currently the most direct gut microbiota-targeted strategy. Mature clinical trials in BPH are lacking, but *in vitro* and animal studies have shown potential. Ferrari et al. established an intestinal barrier-prostate cell coculture model and found that conditioned medium from intestinal models treated with *Bifidobacterium* longum and *Bifidobacterium* psychraerophilum improved BPH-related indices of prostatic cell injury, suggesting potential prostate-protective effects of bifidobacterial probiotics.^83^ Wu et al. found reduced fecal abundance of *Akkermansia muciniphila* (*A. muciniphila*) in patients with BPH and demonstrated in animal models that supplementation with *A. muciniphila* improved the prostatic index and histological changes, suggesting that next-generation probiotics may represent important candidates for microbiota-based BPH therapy.^19^

Prebiotics or polysaccharides with prebiotic-like activity have been more prominently studied in animal models of chronic prostatitis. Liu et al. reported that *P. cocos* polysaccharides alleviated lambda-carrageenan-induced chronic nonbacterial prostatitis in rats and altered gut microbial structure.^38^ Yu et al. further found that metabolites generated by gut microbial fermentation of P. cocos polysaccharides improved the chronic nonbacterial prostatitis phenotype, suggesting that the polysaccharide-gut microbial fermentation product pathway may be an important form of prebiotic-like intervention.^84^

### Dietary and lifestyle interventions

Dietary regulation is the most accessible form of gut microbiota intervention. In a rat model of chronic nonbacterial prostatitis-like inflammation, Konkol et al. found that a diet supplemented with galactoglucomannan-rich fiber altered gut microbial composition and improved prostatitis-related manifestations.^25^ In BPH, Han et al. reported that fermented rape bee pollen improved prostate-related indicators in testosterone-induced BPH rats and modulated dysbiotic gut microbiota, indicating that fermented nutritional products may combine nutritional intervention with microbiota regulation.^85^

Lifestyle exposures also affect gut microbial status in prostatitis. Liu et al. observed that patients with CP/CPPS who consumed alcohol had higher NIH-CPSI scores and abnormalities in gut microbiota and SCFAs, whereas several indicators partially recovered after alcohol discontinuation.^34^ In an animal study, Chen et al. showed that a high-salt diet aggravated EAP and was accompanied by gut dysbiosis, suggesting that limiting alcohol intake and avoiding long-term high-salt diets may help reduce prostatitis-related risk.^86^

### FMT

FMT is currently used mainly in animal experiments to test whether reconstruction of the gut microbiota can modify benign prostatic disease phenotypes. In BPH, Dong et al. found that both sodium butyrate treatment and FMT alleviated prostate enlargement and histological abnormalities in a model of ulcerative colitis-associated prostate enlargement, suggesting that reconstruction of the gut microbiota or supplementation with microbiota-related metabolites may influence the prostatic hyperplasia phenotype.^87^ In chronic prostatitis, FMT studies have focused mainly on EAP models. Du et al. showed that EAP-associated changes in the gut microbiota could influence disease phenotypes through FMT, and subsequent studies further demonstrated, using propionate supplementation and FMT, that gut microbiota modulation can alter prostatitis-related manifestations.^26^^,^^42^ However, no standardized clinical FMT protocol currently exists for patients with BPH or CP/CPPS. Donor screening, safety, efficacy stability, and long-term follow-up remain key barriers to clinical translation.

### Traditional Chinese medicine and natural product interventions

Traditional Chinese medicine formulas, natural products, and plant-derived bioactive compounds are among the fastest-growing areas in microbiota-targeted research for benign prostatic diseases. In animal models of BPH, Wenshenqianlie capsule improved BPH-related parameters and restored gut microbial composition toward a normal state.^88^ Xiaojin Pill improved the BPH phenotype and reshaped the gut microbiota and related metabolic profiles.^89^ Epigallocatechin-3-gallate (EGCG) attenuated BPH progression and regulated dysbiosis associated with Firmicutes predominance.^90^ These studies suggest that traditional Chinese medicine formulas and natural polyphenols may be candidates for microbiota-based adjunctive intervention in BPH, although current evidence remains largely limited to animal experiments. Preliminary human evidence for antioxidant nutraceutical support in BPH has also emerged. Marino et al. conducted a single-arm pilot study in 55 men with elevated PSA levels and mild LUTS who received a multicomponent nutraceutical containing lycopene, sulforaphane, silymarin, glutathione, escin, tryptophan, and green tea extract for 6 months. Reductions in PSA and improvements in patient-reported quality of life were observed, and no serious adverse effects were reported. However, the absence of a control group, the small sample size, and the lack of microbiota or oxidative-stress biomarker measurements preclude conclusions regarding efficacy, mechanism, or microbiota-antioxidant synergy.^91^

Natural products have been studied more extensively in chronic prostatitis. Qiao et al. reported that rapeseed bee pollen alleviated chronic nonbacterial prostatitis and selectively regulated the gut microbiota.^92^ Liu et al. found that astaxanthin improved CP/CPPS-related manifestations by increasing intestinal colonization with *A. muciniphila*.^93^ Tian et al. showed that berberine improved the CP/CPPS phenotype in rats and restored gut microbial diversity and composition.^94^ Ning et al. reported that total tanshinones extract improved CP/CPPS and was accompanied by structural adjustment of the gut microbiota.^28^ Overall, traditional Chinese medicine and natural products offer advantages related to multitarget activity and microecological regulation, but their clinical value still requires validation through standardized preparations, dose optimization, and randomized controlled trials.

### Clinical combination therapies

Based on current clinical evidence, the most translationally relevant strategy is a combination of conventional treatment and microbial restoration, mainly in chronic bacterial prostatitis. Busetto et al. treated chronic bacterial prostatitis with prulifloxacin combined with saw palmetto, *Lactobacillus* sporogenes, and arbutin, and found that the combined regimen produced better symptom improvement and clinical outcomes than antimicrobial therapy alone.^95^ A double-blind randomized study by Chiancone et al. showed that a combination containing cranberry, goji berry, and probiotics (Bifiprost) reduced recurrence of Enterobacteriaceae-associated chronic bacterial prostatitis and improved prostatitis-related symptoms.^96^ Cai et al. reported that *Lactobacillus casei* DG reduced symptomatic recurrences, decreased antibiotic use, and improved quality of life in patients with chronic bacterial prostatitis.^97^ A randomized double-blind trial by Vocca et al. in 2025 further showed that sequential use of *Lactobacillus casei* DG after ciprofloxacin therapy increased the symptom-free interval and promoted recovery of the gut and seminal microbiota.^98^

Phage therapy represents an emerging pathogen-directed option for refractory chronic bacterial prostatitis, particularly when antibiotic-resistant or biofilm-forming organisms are identified. Johri et al. reported successful phage treatment after repeated antibiotic failure in chronic bacterial prostatitis, including a case of recurrent *Escherichia coli* infection. The narrow host range and antibiofilm activity of lytic phages may theoretically permit more selective pathogen eradication and reduce collateral effects on non-target commensal microorganisms compared with broad-spectrum antibiotics.^99^^,^^100^ It is worth noting that these studies were conducted in patients with infection-confirmed chronic bacterial prostatitis (NIH category II), rather than CP/CPPS (NIH category III), and their findings should not be directly extrapolated to CP/CPPS.

In contrast to chronic bacterial prostatitis, direct clinical evidence for microbiota-targeted or microbiota-restorative interventions in CP/CPPS and BPH remains limited. In a study of low-intensity extracorporeal shock wave therapy for CP/CPPS, Kong et al. found that symptom improvement after treatment was accompanied by changes in gut microbial alpha diversity and functional pathways, and that higher baseline abundance of specific pathogenic bacteria was associated with poorer treatment response. These findings suggest that microbial status may influence the efficacy of nonpharmacological therapy.^43^ By contrast, human trial evidence for microbiota-targeted combination therapy in BPH is still lacking. At present, this approach is more appropriately regarded as a potential adjunct to conventional pharmacotherapy, lifestyle intervention, and nutritional management. Future studies should prioritize prospective clinical trials using symptom scores, prostate volume, urinary flow rate, recurrence rate, and fecal microbiota changes as combined endpoints to clarify the true benefits of gut microbiota-targeted therapy in BPH and chronic prostatitis. The principal microbiota-related intervention studies, including their disease populations or experimental models, intervention types, microbiota-related findings, clinical or pathological outcomes, and major limitations, are summarized in Table S2.

## Current challenges and research limitations

Although the gut-prostate axis provides a new perspective for research on benign prostatic diseases, important limitations remain. First, the molecular mechanisms by which the gut microbiota influences the prostatic microenvironment have not been fully elucidated. Existing studies largely infer that SCFAs, bile acid metabolism, systemic low-grade inflammation, Th17/Treg imbalance, and neuroendocrine regulation participate in the development and progression of BPH and CP/CPPS, but direct evidence for key receptors, target cells, and upstream-downstream relationships remains insufficient. Some animal studies indicate that gut dysbiosis can promote immune inflammation and aggravate prostatic inflammation; however, its reproducibility and clinical relevance in human populations require further validation. Second, stable and specific pathogenic microbial biomarkers are still lacking. Although patients with CP/CPPS and BPH have been reported to exhibit microbial structural and metabolic alterations, the differential taxa identified across studies are inconsistent, and causality remains difficult to establish. Clinically, current evidence is derived mainly from single-center, small-sample, cross-sectional studies and is strongly influenced by geography, diet, age, medication use, comorbidities, and sequencing methods, resulting in limited reproducibility. Long-term follow-up is also lacking, making it difficult to determine the dynamic relationships between microbial changes and disease progression, recurrence, and treatment response. In terms of intervention, probiotic therapy lacks standardized strains, doses, and treatment durations, and efficacy varies substantially across individuals and strains. Although FMT can remodel the gut microbial ecology, donor screening, preparation, follow-up costs, risk of infection transmission, and long-term safety require further evaluation. Future research should therefore include multicenter longitudinal cohorts and mechanistic validation studies. Finally, this article is a narrative review and did not employ a preregistered search strategy, formal inclusion and exclusion criteria, or a PRISMA-based study-selection process. The literature was selected to provide representative coverage of the major mechanistic and translational themes. Consequently, some relevant studies may not have been included, and selection bias cannot be completely excluded.

## Conclusions

Overall, the gut microbiota provides a new systems-level perspective for understanding benign prostatic diseases. Current evidence indicates that patients with BPH may exhibit changes in gut microbial structure, enterotype distribution, and metabolic profiles, which are associated with prostate volume, LUTS severity, PSA, postvoid residual urine, and metabolic abnormalities. Patients with CP/CPPS more prominently show reduced microbial diversity, depletion of beneficial taxa, and expansion of potentially proinflammatory taxa, with links to pelvic pain, inflammatory status, and treatment response. The mechanistic implications of the gut microbiota differ between the two conditions. In CP/CPPS, the more direct experimental evidence involves intestinal barrier dysfunction, microbiota-derived metabolites, immune dysregulation, and neural sensitization. In BPH, current evidence mainly supports associations with metabolic inflammation, androgen-sensitive growth signaling, and epithelial-stromal remodeling. Although probiotics, prebiotics, dietary and lifestyle interventions, FMT, and natural products have shown promise, current evidence is still dominated by animal experiments and small-scale studies. Longitudinal cohorts, integrated multi-omics approaches, and mechanistic validation are needed to further clarify the causality and clinical translational value of the gut-prostate axis.

## Acknowledgments

Not applicable.

## Author contributions

Conceptualization, Q.S. and W.F.; data curation, Q.T.; formal analysis, Q.S.; methodology, Q.T. and K.W.; supervision, K.W. and Q.S.; validation, Q.T. and W.F.; writing – original draft and writing – review and editing, Q.S.

## Declaration of interests

The authors declare no competing interests.
